# Lipids by *Yarrowia lipolytica* Strains Cultivated on Glucose in Batch Cultures

**DOI:** 10.3390/microorganisms8071054

**Published:** 2020-07-15

**Authors:** Erdem Carsanba, Seraphim Papanikolaou, Patrick Fickers, Huseyin Erten

**Affiliations:** 1 Food Engineering Department, Faculty of Agriculture, Cukurova University, 01330 Adana, Turkey; ecarsanba@porto.ucp.pt or; 2 Amyris Bioproducts Portugal, Unipessoal, Lda, 4169-005 Porto, Portugal; 3 Department of Food Science& Human Nutrition, Agricultural University of Athens, 11855 Athens, Greece; spapanik@aua.gr; 4 Microbial Processes and Interactions, TERRA Teaching and Research Centre, University of Liège-Gembloux Agro-Bio Tech, B-5030 Gembloux, Belgium; pfickers@uliege.be

**Keywords:** lipid, *Yarrowia lipolytica*, batch culture

## Abstract

Oleaginous microorganisms, such as *Yarrowia lipolytica*, accumulate lipids that can have interesting applications in food biotechnology or the synthesis of biodiesel. *Y. lipolytica* yeast can have many advantages such as wide substrate range usage and robustness to extreme conditions, while under several culture conditions it can produce high lipid productivity. Based on this assumption, in this study, 12 different *Yarrowia lipolytica* strains were used to investigate microbial lipid production using a glucose-based medium under nitrogen-limited conditions in shake-flask cultivations. Twelve wild-type or mutant strains of *Yarrowia lipolytica* which were newly isolated or belonged to official culture collections were tested, and moderate lipid quantities (up to 1.30 g/L) were produced; in many instances, nitrogen limitation led to citric acid production in the medium. Lipids were mainly composed of C16 and C18 fatty acids. Most of the fatty acids of the microbial lipid were unsaturated and corresponded mainly to oleic, palmitic and linoleic acids. Linolenic acid (C18:3) was produced in significant quantities (between 10% and 20%, *wt*/*wt* of dry cell weight (DCW)) by strains H917 and Po1dL.

## 1. Introduction

Microbial lipids or single-cell oils (SCO) contain triacylglycerols (TAGs) (lipids of energy reserve), glycolipids (lipids of membrane structure), phospholipids and sterylesters. Among these lipids, TAGs are the main accumulated component in microbial cells. The synthesis of TAGs by microorganisms begins during the stationary phase of growth with the formation of oil droplets in the cytoplasm, when glucose, other hexoses or polysaccharides or similarly metabolized compounds (i.e., glycerol) are employed as the sole carbon source. The quantity and composition of TAGs accumulated in the cytoplasm of cells depend on the physiology of the microorganism and the culture composition applied, such as the type of carbon and nitrogen sources, as well as physicochemical conditions. In most cases, single-cell oils have a fatty acid composition similar to common plant oils [1,2,3,4,5,6,7]. As a potent oleaginous yeast, *Yarrowia lipolytica* can accumulate lipids to more than 20% of its dry biomass, consisting mostly of unsaturated fatty acids, as in plant oils, and this is of industrial importance in food biotechnology. Beside this, it has been widely used in the production of lipids and lipid-derived compounds such as biodiesel, edible oils or dicarboxylic acids, which are used as building blocks for polymers synthesis [8]. *Y. lipolytica* has many advantages over other oleaginous microorganisms: principally, its high productivity, easier cultivation, wide range of substrate usage (unrefined feedstock and industrial residues) and good tolerance to high substrate concentrations, salt, metal ions and difficult environmental factors such as low and high pH (from 2.5 to 9.0) and a wide range of temperatures (from 18 °C to 32 °C) [6,9,10,11,12].

*Y. lipolytica* has also been identified as a potential citric acid producer. The immediate precursor of cellular lipid accumulation in oleaginous microorganisms is citric acid [13]. Depending on the medium composition and genetic modifications, the production direction can be switched to citric acid, lipid or biomass production. If there is an excess amount of glucose in a medium, cell growth might be followed by significant citric acid production, resulting in a low level of lipid accumulation into cells [14]. Moreover, by the overexpression of specific gene-encoding enzymes in *Y. lipolytica*, citric acid production can also be enhanced. For instance, by the over-expression of the YALI0F00484g gene encoding glycerol kinase and gene YALI0B02948g encoding glycerol-3-P dehydrogenase, glycerol consumption reached 150 g/L within 48 h in bioreactor experiments, and citric acid titer increased in engineered strains 14-fold over control strains [15]. In another study, similarly, by the over-expression of GUT1 (gene encoding glycerol kinase) and GUT2 (gene encoding glycerol-3-P dehydrogenase), 76 g/L of citric acid was produced by an engineered *Y. lipolytica* strain cultivated on crude glycerol at the low pH of 3 after 7 days of batch production [16].

In recent years, the number of publications as well as the economic and technological interest in microbial oil production by *Y. lipolytica* have increased [2,7,8,17,18,19,20]. The production of microbial lipids specifically containing γ-linolenic acid (GLA), eicosapentaenoic acid (EPA), arachidonic acid (ARA) and docosahexaenoic acid (DHA) is currently of increasing interest because these poly-unsaturated fatty acids (FAs) are promising as functional foods and have benefits for human health [8,17,21,22]. Nevertheless, these FAs can only be produced by genetically engineered *Y. lipolytica* strains [17]. On the other hand, a microbial lipid composition similar to cocoa butter substitutes can be produced under several culture mediums and physicochemical conditions [23]. Cocoa butter is known to be an expensive raw material in the chocolate and biscuit industries [24]. Moreover, microbial lipids could be considered as a perfect feedstock for biodiesel production due to their similar composition to several vegetable oils (e.g., rapeseed oil) [25]. Due to all the above benefits, the identification and characterization of microorganisms which are able to produce lipids with a similar composition to high-value fats at high yields is of tremendous financial importance [26]. As a result of this objective, in this study, 12 different *Yarrowia lipolytica* strains were used to investigate microbial lipid production using a glucose-based medium under nitrogen-limited conditions in shake-flask cultivations.

## 2. Material and Methods

### 2.1. Microorganisms, Growth and Culture Conditions

The *Y. lipolytica* strains used in this study were wild type (wt) strains (1) W29 and (2) CBS 6303 and mutant defective strain (3) Po1dL (LEU2 ura3 xpr2), which were maintained in the Microbiology and Molecular Genetics Laboratory (Paris-Grignon, France); strain (4) K57 (wt), which was obtained from the culture collection of the Food Engineering Department, Faculty of Engineering, University of Ankara, Turkey; strain (5) H917 (wt) and mutant defective strains (6) Ain 16 (H222-S4 trs85-1::zeta-URA3), (7) Ain 19 (H222-S4 trs85-2::zeta-URA3), (8) N155 (H222-41 ugt51::zeta-URA3) and (9) Zu 110 (H222-41 pmp47::zeta-URA3) derived from strain H222 (wt), which came from the collection of Institute of Cell Biology, NAS of Ukraine, Ukraine; strains (10) DBVPG 5858 (wt) and (11) DBVPG 4558 (wt), which were obtained from the DBVPG industrial yeast collection, Department of Agricultural, Food and Environmental Science, University of Perugia, Italia; and strain (12) Peggy (wt), which was from the Brewery Industry of Austria.

### 2.2. Shake-Flask Cultivation

For the pre-cultures, single colonies from PDA plates were used to inoculate 250 mL flasks with 50 mL culture medium containing 60 g/L glucose, 0.5 g/L (NH_4_)_2_SO_4_, 0.5 g/L yeast extract, 7 g/L KH_2_PO_4_, 2.5 g/L Na_2_HPO_4_, 1.5 g/L MgSO_4_.7H_2_O, 0.15 g/L CaCl_2_, 0.15 g/L FeCl_3_.6H_2_O, 0.02 g/L ZnSO_4_.7H_2_O and 0.06 g/L MnSO_4_.H_2_O [27]. Cultures were performed at 28 °C with shaking at 185 rpm for 48 or 192 h in an orbital shaker.

The initial cell concentration and pH of the medium were 10^7^ cells/mL and 6.00, respectively. During cultivation, pH was manually regulated between 5.00 and 6.00 by the aseptic addition of a 500–600 µL volume of 5 M KOH within a period of 12 h. Culture samples were collected daily for the determinations of biomass (OD_600_ and dry cell weight), pH, lipids and sugar concentrations.

### 2.3. Methods of Analysis

Dry cell weight (DCW), optical density (OD600) and cell density were used for the determination of biomass. Samples collected from culture medium were centrifuged at 5000 rpm for 15 min and washed twice with distilled water. They were dried at 105 °C until a constant weight for dry cell weight determination. OD_600_ was measured at 600 nm in a spectrometer. Cell density was determined by counting live cells (by staining with methylene blue) in a microscopic chamber (Thoma Lam chamber). The total lipids of the collected biomass ere determined according to the method of Folch, et al. [28]. Cellular lipids were extracted from biomass by using a 2/1 concentration of chloroform/methanol solution. Extracted cellular lipids were esterified for fatty acid profile analysis. n-Heptane and 2 N KOH in methanol were used for the esterification process. Fatty acid methyl esters (FAMEs) were analyzed in a gas chromatograph–mass spectrophotometer (GC–MS) (Hewlett-Packard, USA), equipped with mass spectrometer (MS) detector and an HP-NOWAX (Code: 1909IN-136) column (60 m  ×  0.25 mm  ×  0.25 µm). The injection volume was 1 µL (splitless). Helium was used as a carrier gas with a flow rate of 1 mL/min. The column temperature was started at 120 °C (held for 3 min), then increased at 10 °C/min to 180 °C (held for 19 min) and then at 10 °C/min to 250 °C (held for 19 min). The sugar contents in the culture supernatant were determined by a high-performance liquid chromatograph (HPLC) (Shimadzu brand LC-20AD model), equipped with a Bio-Rad HPX-87H (300 × 7.8 mm) column and an RID detector (RID-10A model). Then, 20 µL of supernatant was injected into the HPLC column and eluted at 50 °C at a flow rate of 0.5 mL/ min using a 5 mM H_2_SO_4_ solution as a mobile phase. The prior analysis of protein content in supernatants was precipitated by the addition of 50 µL of perchloric acid (70%) into 1 ml of sample, which was then centrifuged and filtered through 0.45 µm syringe filters. In total, 20 to 35 dilutions were applied to samples. Each analysis was performed in duplicate.

### 2.4. Statistical Analysis

The data of obtained results were analyzed for analysis of variance (ANOVA) and multiple range tests by using the statistical program SPSS (v.20) for Windows 8 (IBM, Armonk, New York, NY, USA). Principle component analysis (PCA) was performed to simplify the interpretation of the results by using the statistical program PAST (v.3.21) (University of Oslo, Oslo, Norway).

## 3. Results and Discussion

### 3.1. Microbial Lipid Production by Y. lipolytica Strain in Shake-Flask Cultivation

**Figure 1:**
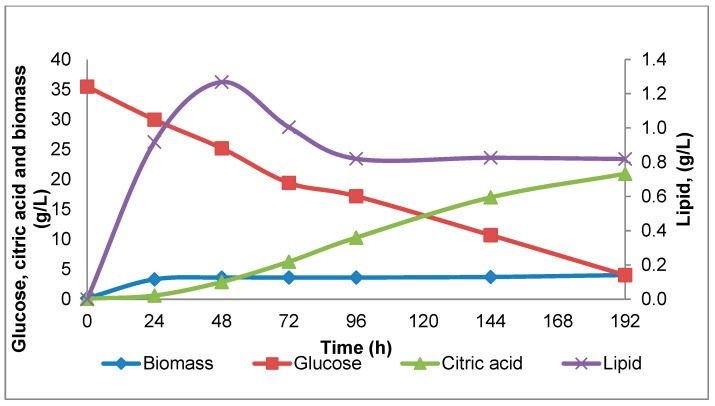
Example of the glucose, biomass, citric acid and lipid content variation for *Y. lipolytica* strain W29 during 192 h shake-flask cultivations.

**Figure 2:**
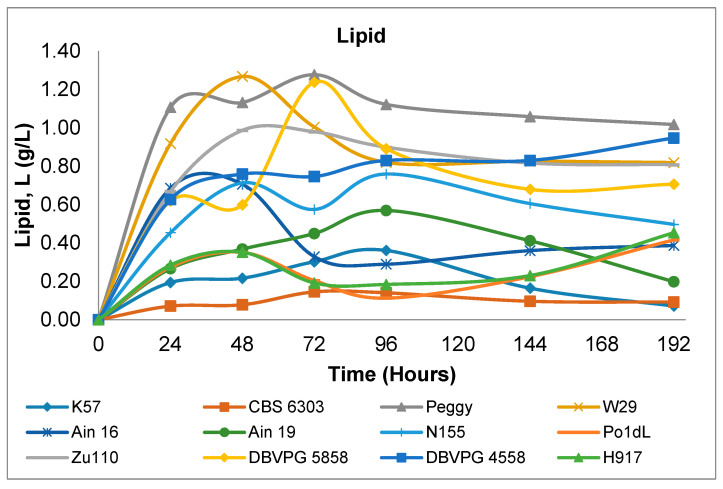
Variations of the lipid content of *Y. lipolytica* strains during 192 h shake-flask cultivation cultures.

An example of the lipid content variation for *Y. lipolytica* strain W29, monitored over eight days of cultivation in shake-flasks, is illustrated in Figure 1. As shown in Figure 2, there is a wide diversity between strains regarding lipid accumulation. The lipid accumulated between 24 to 48 h; at the later stage, lipid accumulation remained constant or even decreased in agreement with previous results of Sabra, et al. [29] (Figure 1). In general, the higher lipid content varied between 0.4 to 1.30 g/L on the second or third day of cultivation. In some cases, lipid accumulation was very low, especially for the strains K57 and CBS 6303, whereas higher quantities were exhibited for strains Peggy, W29 and DBVPG 5858.

When glucose, glycerol or similarly catabolized compounds are employed as carbon sources of *Y. lipolytica* in batch nitrogen-limited cultures, there are three different types of lipid accumulation metabolism that are observed. In the first type—called typical oleaginous metabolism—high quantities of lipid are accumulated after nitrogen exhaustion in the medium with a simultaneously lower amount of extracellular metabolite production, such as citric acid (CA) and polyols [30,31]. In the second type—referred to as atypical oleaginous metabolism—lipids are stored at the beginning of cultivation after nitrogen depletion in the medium, and at a later stage, citric acid production occurs and continues uninterrupted, accompanied by decreasing lipid concentration [25,32,33,34]. The third type of metabolism is an atypical metabolism, in which lipids in yeast cells accumulate at slow rates without degradation and with the simultaneous secretion of citric acid [29]. According to this assumption, as can be seen in Figure 2, while strains Peggy and DVPG 5858 showed a typical oleaginous metabolism, strains CBS 6303 and K57 displayed an atypical metabolism. Moreover, the third type of atypical oleaginous metabolism was observed for strains Zu110 and W29, in which lipid accumulation occurred with continuous citric acid production (Table 1 and Table 2).

**Figure 3:**
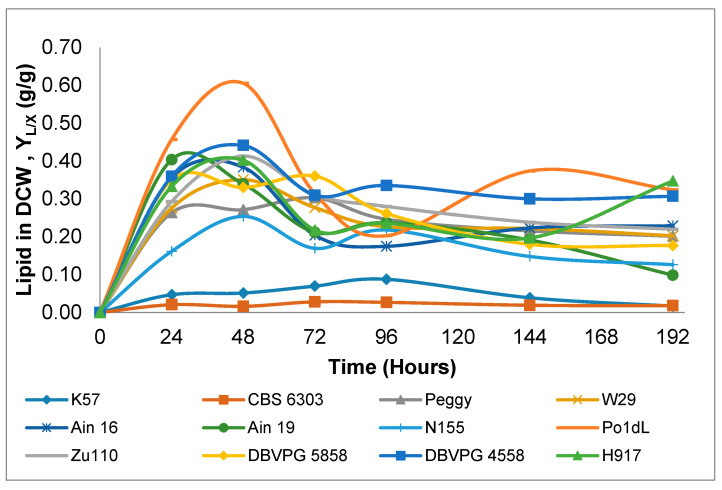
Variations of the lipid in dry cell weight (DCW) (*g*/*g*) of *Y. lipolytica* strains during 192 h shake-flask cultivations.

Generally, the maximum lipid in DCW (*g*/*g*) values—rather than lipid content values—are preferred to evaluate the lipid-producing capacity of oleaginous microorganisms. The maximum lipid in DCW (*g*/*g*) values and lipid contents of 12 *Y. lipolytica* strains were determined. Strains K57 and CBS 6303 obviously did not display lipid-producing characteristics, with lipid in DCW (*g*/*g*) values of 0.09 and 0.03, which were very low quantities when compared with the other strains (Table 1). This indicated that the cultivation of these two *Y. lipolytica* strains in a glucose-based nitrogen-limited environment did not exhibit de novo TAG synthesis and thus lipid accumulation, as the percentage of intracellular lipid content for de novo accumulation should be more than 20%. This behavior can be explained by a switch of metabolic pathways toward the synthesis of citric acid instead of lipid de novo synthesis and accumulation [32]. However, strains Po1dL, DBVPG 4558, Zu110, Ain 19, Ain 16 and H917 had higher lipid in DCW (*g*/*g*) values, ranging between 0.39 and 0.61 *g*/*g*, respectively. In addition, the variation of lipid in DCW (*g*/*g*) of the 12 tested strains is illustrated in Figure 3, in which higher values are generally observed on the second day of cultivation and later remained constant or even decreased. This characteristic of strains was also observed in other studies reported in the literature [25,29,32,35]. Dourou, et al. [5] demonstrated that the lipid metabolism of *Y. lipolytica* during glucose consumption in shake-flask cultivation occurs in three different phases; i.e., balanced growth, oleaginous and lipid turnover phases. The balanced growth phase concluded with the exhaustion of nitrogen in the growth medium. The oleaginous phase began after the depletion of nitrogen and finished with glucose in the medium. The final phase, the “lipid turnover phase”, occurred after glucose depletion in the cultivation medium. Relatively high amounts of lipid were stored from glucose during the transition from the early oleaginous to late oleaginous phase. As can be seen in Figure 3, the time interval from 0 to 24 h of cultivation shows a balanced growth phase, while the interval of 24 to 48 h (some cases until 72 h) indicates the oleaginous phase, and the sixth day of cultivation can be inferred as the lipid turnover phase of most strains (except strain H917).

The maximum lipid in DCW (*g*/*g*) values and lipid contents varied from 0.03 to 0.61 *g*/*g* and 0.15 to 1.28 g/L, respectively, and were remarkably high values when compared with the results obtained by *Y. lipolytica* in shake-flask cultivations using glucose-based media reported in other studies. For instance, maximum lipid in DCW (*g*/*g*) values lower than 0.04 *g*/*g* were obtained for strains ACA-YC 5033, ACA-YC 5029 and W29 [32]. In the present study, only strains K57 and CBS 6303 exhibited lipid in DCW (*g*/*g*) values lower than 0.09 *g*/*g*. Moreover, strain W29 generated a higher lipid in DCW (*g*/*g*) value, at 0.35 *g*/*g*, than reported by Papanikolaou, et al. [32]. Similar results were also reported by Papanikolaou, et al. [35] with regards to the lipid in DCW (*g*/*g*) of *Y. lipoytica* ACA-DC 50109, which were found to range between 0.05 and 0.12 *g*/*g* for different glucose concentrations (34 to 150 g/L glucose concentrations) during shake-flask cultivations. Moreover, in another study with the same strain, Dourou, et al. [5] reported a maximum lipid in DCW (*g*/*g*) value of 0.33 *g*/*g* from a glucose concentration of 40 g/L in the late oleaginous phase of a shake-flask cultivation. Recently, Sabra, et al. [29] also reported a lipid in DCW (*g*/*g*) value of 0.12 *g*/*g* from a starting glucose concentration of 50 g/L using the same strain in shake-flask cultivation. Despite these low lipid in DCW (*g*/*g*) values produced by wild-type *Y. lipolytica* strains, a metabolically engineered strain of *Y. lipolytica*, YL-ad9, was capable of accumulating a lipid content of 67% (*g*/*g*) from glucose in a fed-batch cultivation [36]. Although this value was found to be close to the 0.61 *g*/*g* obtained for strain Po1dL in the current study, the final biomass and lipid content obtained for this strain were very low (1.29 and 0.42 g/L, respectively).

### 3.2. Composition of Microbial Lipids Produced by Y. lipolytica Strains

**Figure 4:**
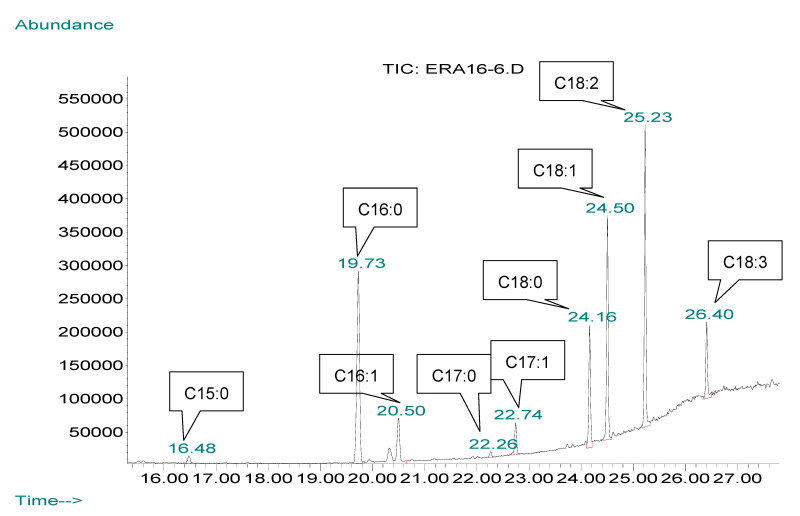
Example of a GC–MS (Gas chromatography–mass spectrophotometry) chromatogram of fatty acids of Ain 16 strain in the late stationary phase (C15:0—pentadecanoic acid, C16:0—palmitic acid, C16:1—palmitoleic acid, C17:0—margaric acid, C17:1—heptadecenoic acid, C18:0—stearic acid, C18:1—oleic acid, C18:2—linoleic acid, C18:3—linolenic acid).

It was reported that lipids produced by *Y. lipolytica* from glucose were mainly formed from polar fractions such as phospholipids, with fatty acid (FA) concentrations of Δ9C18:1 43 ± 4% *wt*/*wt* (weight/weight), C18:0 7 ± 3% *wt*/*wt*, C16:0 16 ± 5% *wt*/*wt*, Δ 9,12C18:2 18 ± 4% *wt*/*wt* and Δ 9C16:1 7 ± 4% *wt*/*wt* [35]. As can be observed in the FA composition (Table 3), the lipid produced by *Y. lipolytica* has an unsaturated characteristic due to the high concentrations of unsaturated FAs (C18:1, C18:2 and C16:1 are approximately 68% of the total cellular lipid). The ratio of unsaturated to saturated FAs was found to be 3.5 in the same study [35]. This trend of FA composition can also be deduced from the present results, in that synthesized FAs mostly range from highest to lowest concentrations in the following order: C18:1, C16:0, C18:2, C16:1, C18:0, C18:3 and others in a small amount (C17:1, C:17:0 and C15:0) (Table 3 and Figure 4).

On the basis of the lipid in DCW (*g*/*g*) graph (Figure 3), the FA composition of each strain was examined at two different stages of the early stationary phase (48 h after inoculation) and late stationary phases (144 h after inoculation). The early stationary phase is a time period in which lipid accumulation increases; in contrast, during the late stationary phase—referred to as the lipid turnover period—accumulated lipids and stored lipids inside cells decrease [32]. The results of FA composition are presented in Table 3, in which oleic acid (C18:1), palmitic acid (C16:0) and linoleic acid (C18:2) are the most abundant FAs produced inside the cell of all examined *Y. lipolytica* strains. Moreover, linolenic acid (C18:3) was produced in significant quantities (between 10 and 20%, *wt*/*wt*), especially by strains H917 and Po1dL. Other FAs, such as C15:0, C17:0 and C17:1, were also found in small amounts for all the strains except strain H917 (approximately 17% of C17:0 and 6% of C17:1 were found). Another point of view regarding FA composition involves the FA profile between the early and late stationary phases, as a slight change in FA profile was obtained for strains Ain 16, Ain 19, DBVPG 4558, K57 and N155, whereas drastic differences were found for strains CBS 6303, DBVPG 5858, H917, Po1dL, Peggy, W29 and Zu110. For instance, the proportions of C16:0, C18:2 and C18:3 in the early stationary phase, increased whereas they decreased for C16:1, C18:0 and C18:1 in the late stationary phase. Generally, a conversion into the group of C18 chains (C18:0, C18:1, C18:2 and C18:3) and C16 chains (C16:0 and C16:1) was observed at different stationary phases with varying concentrations.

In addition, the ratio of unsaturated to saturated fatty acids (UFAs/SFAs) indicated that lipids produced in the early stationary phase are, in general, characterized as more unsaturated than those in the late stationary phase, as the ratio of UFAs/SFAs decreased; i.e., the unsaturated profile decreased through the late stationary phase. Among the FA profiles, only strains Peggy and Po1dL in the stationary phase, strain H917 in the early phase and strain Zu110 in the late stationary phase showed a saturated profile.

When the results of the FA composition from this study are compared to those from the literature [29,32,35], it can be observed that similar FA compositions were obtained. The most dominant FAs in cellular lipid produced by all strains were oleic, palmitic, linoleic and stearic acids. The only difference was the linolenic acid content, which in some cases was higher than previous results reported in the literature. Moreover, the cellular FA composition produced by strain W29 during cultivation on 30 g/L glucose-based media investigated by Papanikolaou, et al. [32] was different to this study in that the concentration of C16:0 (around 19% in the work performed by Papanikolaou, et al. [32] and 25% in this study) was lower but C18:1 (around 55% in the work performed by Papanikolaou, et al. [32] and 39% in this study) was higher.

**Figure 5:**
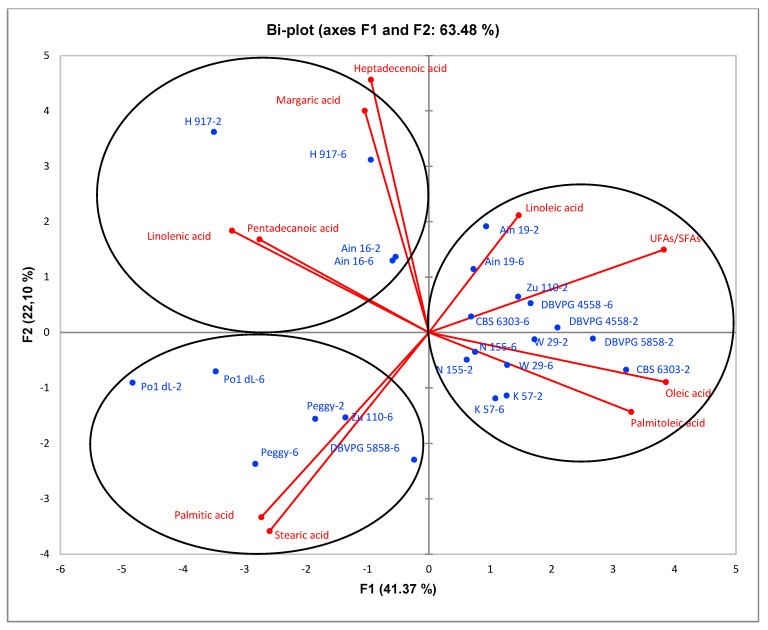
Bi-plot of principal components analysis (PCA) regarding the fatty acid composition of *Y. lipolytica* strains in shake-flask cultivations.

PCA analysis was performed to characterize the fatty acid composition generated by 12 *Y. lipolytica* strains in shake-flask cultures. For each strain, two samples, collected on the second and sixth day of cultivation, were considered. In general, on the second day of cultivation, the lipid in DCW (*g*/*g*) value was higher; on the sixth day of cultivation, lipid turnover was observed. Figure 5 shows a bi-plot of PCA which explains overall 63.48% of the total variance, composed of 41.37% of F1 and 22.10% of F2. Three different distinct groups of strains on the bi-plot are notable due to their fatty acid composition. In general, fatty acids of palmitic (C16:0), stearic (C18:0), linoleic (C18:2), linolenic (C18:3), palmitoleic (C16:1), oleic (C18:1), pentadecanoic (C15:0), margaric (C17:0) and heptadecenoic acids (C17:1) were observed. The first group was generated by strains Ain19, CBS 6303, W29, K57, DBVPG 4558, N155, Zu110-2 and DBVPG 5858-2, characterized by palmitoleic, oleic and linoleic acids and UFAs/SFAs; the second group consisted of strains H917 and Ain16, identified by heptadecenoic, margaric, pentadecanoic and linolenic acids. Finally, the third group was formed by strains Po1dL, Peggy, Zu110-6 and DBVPG 5858-6, differentiated by palmitic and stearic acids. Considering the PCA results, it can be said that most of the strains were characterized by the property of unsaturation; i.e., oleic, palmitoleic and linoleic acids. However, strains Peggy, Po1dL and sixth-day samples of Zu110 and DBVPG 5858 showed more saturated characteristics consisting of a high level of palmitic and stearic acids. In addition, linolenic acid was generated to a greater degree by strains H917 and Ain16.

The lipid content and fatty acid profile synthesized by *Y. lipolytica* are also dependent on the feedstock used. Different types of cheap carbon sources were employed in lipid production via *Y. lipolytica* cultivation. For instance, 15–25% (*wt*/*wt*) of lipids, consisting of major fatty acids such as oleic acid and palmitic acid, were produced from olive mill wastewater-based media [37]. Moreover, in a study of microbial lipid production from industrial derivative of tallow, 52.0% of lipid was accumulated in *Y. lipolytica* strain ACA-DC 50109 [38]. In the same study, the fatty acid composition was found to be rich in saturated fatty acids, mainly stearic acid (78% *wt*/*wt*) and palmitic acid (17% *wt*/*wt*). In addition, glycerol-based media (crude glycerol) have been considered as a cheap feedstock, and in nitrogen-limited conditions, lipids accumulated in *Y. lipolytica* cultıvated on industrial glycerol contained higher amounts of oleic, linoleic, palmitic and palmitoleic acid [39]. In another study, a similar fatty acid profile to *Y. lipolytica* strain S6 cultivated on 25 g/L pure glycerol or glycerol fraction was obtained [12]. Leiva-Candia, et al. [40] and Qin, et al. [3] also reviewed potential agro-industrial waste utilization using oleaginous yeast for lipid production and explained that *Y. lipolytica* can produce different amounts of lipid with varying fatty acid compositions depending on the carbon source chosen or culture conditions employed. The fatty acid profiles obtained by *Y. lipolytica* cultivation on different agro-industrial feedstocks reported in these studıes were similar to vegetable oils; e.g., palm oil. Results from this study also showed that, in general, the major fatty acids of oleic, palmitic, linoleic, stearic and palmitoleic acid, corresponding to the fatty acids profile of vegetable oils, were produced by different *Y. lipolytica* strains during cultivation on glucose-based media. This lipid, which is similar to the fatty acid profile of vegetable oils, produced by *Y.lipolytica* grown in glucose-based media has the potential to be used in the sustainable and renewable biodiesel industry.

## 4. Conclusions

Among the examined strains, of the 12 strains, ten *Y. lipolytica* strains exhibited an oleaginous property, producing more than 20% (g lipid/g dry cell weight) of the lipid in dry cell weight value (in some cases, 45% of lipid in DCW)—two strains did not exhibit this property (K57 and CBS 6303). According to results, lipids were produced more in the early stationary phase than late stationary phase of cultivation. PCA analysis allowed us to distinguish three different groups of strains according to their FA profile. Most of the tested strains generated in the first group were characterized by an unsaturated profile, while a saturated profile was observed for the second group (strains Peggy and Po1dL). Moreover, the third group (consisting of strains H917 and Ain16) consisted of unusual fatty acids such as linolenic, pentadecanoic, heptadecenoic and margaric acids. This is the first study that has shown that *Y. lipolytica* strains can be differentiated according to their FA profile.

## Figures and Tables

**Table 1:** Values of DCW (g/L), consumed glucose (g/L), maximum lipid content (g/L) and lipid in DCW (*g*/*g*) for *Y. lipolytica* strains in shake-flask cultivations (N = 2).

	Incubation Time (h)	DCW (g/L)	Consumed Glucose (g/L)	Maximum Lipid Content, L_m_ (g/L)	Maximum Lipid in DCW (*g*/*g*) Y_mL/X_ (*g*/*g*)
Po1dL	192	1.29 ± 0.05	8.19 ± 0.10	0.42 ± 0.01 ^c^	
	48	0.58 ± 0.06	0.09 ± 0.01		0.61 ± 0.06 ^e^
DBVPG 4558	192	3.09 ± 0.01	23.61 ± 0.13	0.95 ± 0.01 ^h^	
	48	1.72 ± 0.34	7.22 ± 0.18		0.45 ± 0.08 ^d^
Zu110	48	2.39 ± 0.35	7.17 ± 0.09	0.99 ± 0.01 ^i^	0.42 ± 0.06 ^cd^
Ain19	96	2.40 ± 0.13	5.71 ± 1.21	0.57 ± 0.00 ^e^	
	24	0.66 ± 0.01	1.07 ± 0.33		0.41 ± 0.01 ^cd^
H917	192	1.31 ± 0.08	9.64 ± 1.30	0.46 ± 0.01 ^d^	
	48	0.88 ± 0.01	1.81 ± 0.10		0.41 ± 0.01 ^cd^
Ain16	48	1.84 ± 0.08	3.76 ± 0.11	0.71 ± 0.01 ^f^	0.39 ± 0.02 ^cd^
DBVPG 5858	72	3.44 ± 0.40	10.93 ± 1.07	1.24 ± 0.01 ^j^	0.36 ± 0.04 ^bcd^
W29	48	3.62 ± 0.06	10.29 ± 0.88	1.27 ± 0.02 ^k^	0.35 ± 0.00 ^bcd^
Peggy	72	4.22 ± 0.13	10.13 ± 0.42	1.28 ± 0.01 ^k^	0.31 ± 0.01 ^bc^
N155	96	3.49 ± 0.05	11.21 ± 1.61	0.76 ± 0.00 ^g^	
	48	2.81 ± 0.01	3.88 ± 0.29		0.25 ± 0.00 ^b^
K57	96	4.12 ± 0.18	22.74 ± 2.33	0.36 ± 0.00 ^b^	0.09 ± 0.00 ^a^
CBS 6303	72	5.16 ± 0.11	13.88 ± 2.13	0.15 ± 0.01 ^a^	0.03 ± 0.00 ^a^

Results are the mean of two replications ± standard deviations; ^a,b,c,d,e,f,g,h,I,j,k^ Various superscript letters in the same row demonstrate significant differences at the 0.05 level among samples (*p* < 0.05).

**Table 2:** Kinetic values for *Y. lipolytica* strains in shake-flask cultivations (N = 2).

	K57	CBS 6303	Peggy	W29	Ain 16	Ain 19	N155	Po1dL	Zu110	DBVPG 5858	DBVPG 4558	H917
**X_max_**	4.34 ± 0.26	5.23 ± 0.29	5.05 ± 0.10	4.04 ± 0.06	1.93 ± 0.09	2.40 ± 0.13	4.09 ± 0.20	1.29 ± 0.05	3.68 ± 0.05	4.00 ± 0.01	3.09 ± 0.01	1.31 ± 0.08
**Y_X/S_**	0.25 ± 0.04	0.27 ± 0.04	0.29 ± 0.00	0.13 ± 0.00	0.53 ± 0.07	0.42 ± 0.05	0.27 ± 0.01	0.16 ± 0.00	0.13 ± 0.00	0.21 ± 0.00	0.13 ± 0.00	0.39 ± 0.11
**Y_C/S_**	0.43 ± 0.05	0.58 ± 0.08	0.39 ± 0.01	0.67 ± 0.01	0.00 ± 0.00	0.00 ± 0.00	0.55 ± 0.00	0.21 ± 0.01	0.75 ± 0.00	0.27 ± 0.00	0.69 ± 0.01	0.15 ± 0.07
**Y_L/S_**	0.02 ± 0.00	0.007 ± 0.00	0.06 ± 0.00	0.03 ± 0.00	0.19 ± 0.02	0.10 ± 0.02	0.04 ± 0.00	0.05 ± 0.00	0.03 ± 0.00	0.04 ± 0.00	0.04 ± 0.00	0.14 ± 0.05
**Q_C_**	0.10 ± 0.00	0.12 ± 0.01	0.03 ± 0.00	0.11 ± 0.00	0.00 ± 0.00	0.00 ± 0.00	0.06 ± 0.00	0.01 ± 0.00	0.11 ± 0.00	0.03 ± 0.00	0.08 ± 0.00	0.003 ± 0.00
**L_max_**	0.36 ± 0.00	0.15 ± 0.00	1.28 ± 0.01	1.27 ± 0.02	0.71 ± 0.01	0.57 ± 0.00	0.76 ± 0.00	0.42 ± 0.01	0.99 ± 0.01	1.24 ± 0.01	0.95 ± 0.01	0.45 ± 0.01
**Y_X/S_**	0.18 ± 0.01	0.37 ± 0.12	0.42 ± 0.03	0.35 ± 0.00	0.49 ± 0.07	0.42 ± 0.05	0.31 ± 0.05	0.16 ± 0.00	0.33 ± 0.06	0.31 ± 0.05	0.13 ± 0.00	0.39 ± 0.11
**Y_C/S_**	0.51 ± 0.05	0.48 ± 0.15	0.20 ± 0.01	0.28 ± 0.00	0.00 ± 0.00	0.00 ± 0.00	0.39 ± 0.06	0.21 ± 0.01	0.32 ± 0.01	0.14 ± 0.01	0.69 ± 0.01	0.15 ± 0.07
**Y_L/S_**	0.02 ± 0.00	0.01 ± 0.00	0.13 ± 0.01	0.12 ± 0.00	0.19 ± 0.02	0.10 ± 0.02	0.07 ± 0.01	0.05 ± 0.00	0.14 ± 0.01	0.11 ± 0.01	0.04 ± 0.00	0.14 ± 0.05
**Q_C_**	0.12 ± 0.01	0.09 ± 0.00	0.03 ± 0.00	0.06 ± 0.00	0.00 ± 0.00	0.00 ± 0.00	0.05 ± 0.00	0.01 ± 0.00	0.05 ± 0.00	0.02 ± 0.00	0.08 ± 0.00	0.003 ± 0.00
**C_max_**	21.29 ± 0.02	24.67 ± 0.78	6.71 ± 0.06	20.94 ± 0.02	0.47 ± 0.06	0.79 ± 0.05	9.28 ± 0.03	1.68 ± 0.02	20.47 ± 0.01	5.08 ± 0.02	16.21 ± 0.05	0.48 ± 0.05
**Y_X/S_**	0.12 ± 0.00	0.14 ± 0.01	0.29 ± 0.00	0.13 ± 0.00	0.16 ± 0.01	0.21 ± 0.05	0.23 ± 0.01	0.16 ± 0.00	0.13 ± 0.00	0.21 ± 0.00	0.13 ± 0.00	0.39 ± 0.11
**Y_C/S_**	0.59 ± 0.01	0.67 ± 0.03	0.39 ± 0.01	0.67 ± 0.01	0.04 ± 0.00	0.08 ± 0.01	0.54 ± 0.02	0.21 ± 0.01	0.75 ± 0.00	0.27 ± 0.00	0.69 ± 0.01	0.15 ± 0.07
**Y_L/S_**	0.002 ± 0.00	0.003 ± 0.00	0.06 ± 0.00	0.03 ± 0.00	0.04 ± 0.00	0.02 ± 0.00	0.03 ± 0.00	0.05 ± 0.00	0.03 ± 0.00	0.04 ± 0.00	0.04 ± 0.00	0.14 ± 0.05
**Q_C_**	0.11 ± 0.00	0.13 ± 0.00	0.03 ± 0.00	0.11 ± 0.00	0.002 ± 0.00	0.004 ± 0.00	0.05 ± 0.00	0.01 ± 0.00	0.11 ± 0.00	0.03 ± 0.00	0.08 ± 0.00	0.003 ± 0.00

(Xmax—maximum biomass (g/L), Cmax—maximum citric acid (g/L), Lmax—maximum lipid (g/L), Y_X/S_—biomass yield (*g*/*g*), Y_C/S_—citric acid yield (*g*/*g*), Y_L/S_—lipid in DCW (*g*/*g*) (*g*/*g*), Qc—volumetric productivity of citric acid (g/L/h)).

**Table 3:** Fatty acid composition of total lipids produced by *Y. lipolytica* strains during early (second day of cultivation) and late stationary (sixth day of cultivation) growth phases on glucose-based media. UFAs/SFAs: ratio of unsaturated to saturated fatty acids.

		Fatty Acid Composition %									UFAs/SFAs	Lipid in DCW (*g*/*g*)Y_L/X_, *g*/*g*
Strain	Growth Phase	15:0	16:0	16:1	17:0	17:1	18:0	18:1	18:2	18:3		
Ain 16	Early Stationary	0.9	26.9	7.2	0.7	3.3	10.5	17.8	25.1	7.4	1.6	0.4
	Late Stationary	1.0	27.1	7.1	0.7	3.3	10.6	18.8	24.3	7.0	1.5	0.2
Ain 19	Early Stationary	0.9	23.4	9.3	1.2	4.3	8.0	24.6	24.3	4.0	2.0	0.3
	Late Stationary	1.0	24.2	9.1	1.0	3.2	9.5	27.6	21.1	3.3	1.8	0.2
CBS 6303	Early Stationary		21.5	9.3			8.8	47.0	13.4		2.3	0.0
	Late Stationary		31.8	5.7			2.8	21.7	28.1	9.9	1.9	0.0
DBVPG 4558	Early Stationary	0.6	20.6	9.4	3.5	0.5	8.6	43.6	11.5	1.6	2.0	0.4
	Late Stationary	0.8	20.4	8.7	5.6	0.9	8.5	41.6	12.3	1.2	1.8	0.3
DBVPG 5858	Early Stationary	0.7	23.0	12.6			5.9	38.6	15.5	3.6	2.4	0.3
	Late Stationary	0.2	29.5	11.7			19.9	24.9	7.8	6.0	1.0	0.2
H917	Early Stationary	1.1	23.4	2.1	17.2	6.1	12.2	14.6	9.0	14.2	0.8	0.4
	Late Stationary	0.5	17.1	4.3	21.8	1.7	6.6	22.5	13.6	11.8	1.2	0.2
K57	Early Stationary		26.7	7.4	3.0		11.8	38.5	12.6		1.4	
	Late Stationary	0.2	26.5	7.6			13.6	35.8	15.8	0.5	1.5	
N155	Early Stationary	0.7	26.6	8.8	0.9	1.5	12.8	34.3	13.9	0.5	1.4	0.2
	Late Stationary	0.6	28.6	8.7	0.6	1.2	10.1	30.7	18.8	0.7	1.5	0.1
Po1dL	Early Stationary	1.5	37.2	2.3			19.0	10.0	10.0	19.9	0.7	0.6
	Late Stationary	1.1	34.0	2.6			16.4	15.4	12.2	18.3	0.9	0.4
Peggy	Early Stationary	0.8	37.4	6.7	2.5	0.8	15.5	22.8	10.4	2.9	0.8	0.3
	Late Stationary	1.0	31.6	6.4	0.4	0.5	30.4	17.3	8.8	3.5	0.6	0.2
W29	Early Stationary	0.2	25.2	8.3		0.8	8.3	34.5	18.6	4.1	2.0	0.3
	Late Stationary	0.3	23.1	6.3	0.3	0.4	13.7	38.8	16.1	1.1	1.7	0.2
Zu110	Early Stationary	0.8	21.9	6.2	0.6	1.0	9.9	35.5	23.2	0.9	2.0	0.4
	Late Stationary	0.4	28.9	5.4	0.3	1.1	25.6	20.0	18.2		0.8	0.2

C15:0—pentadecanoic acid, C16:0—palmitic acid, C16:1—palmitoleic acid, C17:0—margaric acid, C17:1—heptadecenoic acid, C18:0—stearic acid, C18:1—oleic acid, C18:2—linoleic acid, C18:3—linolenic acid.
